# Glial Fibrillary Acidic Protein as Biomarker Indicates Purity and Property of Auricular Chondrocytes

**DOI:** 10.1089/biores.2019.0058

**Published:** 2020-03-03

**Authors:** Satoru Nishizawa, Sanshiro Kanazawa, Yuko Fujihara, Yukiyo Asawa, Satoru Nagata, Motohiro Harai, Atsuhiko Hikita, Tsuyoshi Takato, Kazuto Hoshi

**Affiliations:** ^1^ Translational Research Center, The University of Tokyo Hospital, Tokyo, Japan.; ^2^ Department of Cell and Tissue Engineering (Fujisoft) and Graduate School of Medicine, The University of Tokyo, Tokyo, Japan.; ^3^ Department of Sensory and Motor System Medicine, Graduate School of Medicine, The University of Tokyo, Tokyo, Japan.; ^4^ NAGATA Microtia and Reconstructive Plastic Surgery Clinic, Toda City, Japan.; ^5^ FUJISOFT Tissue Engineering Co., Ltd., Yokohama-shi, Japan.

**Keywords:** GFAP, chondrocyte, auricular

## Abstract

Instead of the silicone implants previously used for repair and reconstruction of the auricle and nose lost due to accidents and disease, a new treatment method using tissue-engineered cartilage has been attracting attention. The quality of cultured cells is important in this method because it affects treatment outcomes. However, a marker of chondrocytes, particularly auricular chondrocytes, has not yet been established. The objective of this study was to establish an optimal marker to evaluate the quality of cultured auricular chondrocytes as a cell source of regenerative cartilage tissue. Gene expression levels were comprehensively compared using the microarray method between human undifferentiated and dedifferentiated auricular chondrocytes to investigate a candidate quality control index with an expression level that is high in differentiated cells, but markedly decreases in dedifferentiated cells. We identified glial fibrillary acidic protein (GFAP) as a marker that decreased with serial passages in auricular chondrocytes. GFAP was not detected in articular chondrocytes, costal chondrocytes, or fibroblasts, which need to be distinguished from auricular chondrocytes in cell cultures. GFAP mRNA expression was observed in cultured auricular chondrocytes, and GFAP protein levels were also measured in the cell lysates and culture supernatants of these cells. However, GFAP levels detected from mRNA and protein in cell lysates were significantly decreased by increases in the incubation period. In contrast, the amount of protein in the cell supernatant was not affected by the incubation period. Furthermore, the protein level of GFAP in the supernatants of cultured cells correlated with the *in vitro* and *in vivo* production of the cartilage matrix by these cells. The productivity of the cartilage matrix in cultured auricular chondrocytes may be predicted by measuring GFAP protein levels in the culture supernatants of these cells. Thus, GFAP is regarded as a marker of the purity and properties of cultured auricular chondrocytes.

## Introduction

The external nose and external ear are organs maintaining their shape with cartilage, and repair and reconstruction are performed when these are deformed or lost due to congenital anomalies or trauma, in which a frame prepared with silicone, costal cartilage, and other biomaterials or transplants is transplanted to the affected region. Since silicone is an artificial material, it may have a negative influence after transplantation. Costal cartilage is a superior onlay graft, but its collection is very invasive and it has a risk of development of pneumothorax and thoracic deformity. Moreover, a frame made of costal cartilage may be deformed after transplantation.^1,2^ A new treatment strategy applying tissue engineering has been attracting attention as a method to overcome these problems. Chondrocytes are collected from the cartilage tissue of patients, and cartilage is regenerated by culturing and processing these cells and transplanting them into a defective site.^3^ This treatment may enable impaired cartilage tissue to recover its original structure and function, which cannot be achieved by conventional treatment methods. However, many obstacles remain before the application of this method to clinical settings. One of which is the establishment of a method to evaluate the quality of chondrocytes and the cell source of regenerated cartilage.^4^

The differentiation characteristics of cells need to be assessed as the initial step in the evaluation of the quality of auricular chondrocytes. Physiological auricular chondrocytes secrete type II collagen and proteoglycan and form a specific cartilage matrix. When auricular chondrocytes are grown in cultures, these characteristics are lost and, inversely, type I collagen secretion is enhanced. This phenomenon is termed dedifferentiation.^5–7^ Since the number of auricular chondrocytes that may be collected from a patient to prepare regenerated cartilage is limited, this number needs to be increased in cultures. However, when auricular chondrocytes excessively proliferate, the progression of dedifferentiation is accelerated. Furthermore, the production of the cartilage matrix decreases in excessively dedifferentiated auricular chondrocytes; therefore, regenerative cartilage using these cells as a material may have a poor cartilage matrix.^8^ Based on these findings, an index to confirm whether cultured auricular chondrocytes are excessively dedifferentiated is needed. A large number of auricular chondrocytes are necessary to prepare tissue-engineered cartilage with a size applicable for repair and reconstruction of the external nose and auricle, but the collectable amount of cartilage is limited. Thus, it is necessary to amplify auricular chondrocytes by 2D passage culture. However, since auricular chondrocytes are isolated from cartilage excised from the body, auricular chondrocytes may be mixed with other types of cells derived from the perichondrium and connective tissue surrounding the cartilage tissue, such as fibrous tissue. Since these cells grow faster than auricular chondrocytes, although fewer cells are contaminated, the dominant cell type after 2D passage culture may be different from that at the time of auricular chondrocyte isolation. Therefore, an index to confirm that cells are auricular chondrocytes is also needed.

In the field of regenerative medicine, the gene expression levels of *COL2A1, ACAN, SOX9*, and *SSEA4* and the positive rates of CD44 and CD54 surface antigens have been reported as indices to measure the differentiation degree of chondrocytes.^9,10^ However, these indices are insufficient because their specification as indices to evaluate the quality of auricular chondrocytes as a material of regenerated cartilage has not yet been established. Moreover, an index has not been developed to judge the presence of cells other than target auricular chondrocytes, such as fibroblasts contained in the perichondrium and surrounding fibrous tissue.

The objective of this study was to establish an optimal index to evaluate the quality and purity of cultured auricular chondrocytes, which are clinically used as a cell source of regenerative cartilage for the correction of facial deformities.^11^ In this study, gene expression levels were comprehensively compared between human differentiated and dedifferentiated auricular chondrocytes using a microarray method to identify a candidate index with an expression level that is higher in differentiated cells, but markedly decreases in dedifferentiated cells. Changes in the protein level of this candidate factor were then examined in cell lysates and culture supernatants, followed by the verification of its localization in native auricular cartilage tissue and expression in cell types other than auricular chondrocytes. Furthermore, the relationship between the protein level of the candidate factor in human auricular chondrocytes and the *in vitro* production of the cartilage matrix was investigated. The applicability of the candidate factor to the quality control of cultured human auricular chondrocytes was confirmed by the relationship between the protein level of the candidate factor in human auricular chondrocytes and the quality of regenerative cartilage produced by these cells *in vivo*.

## Materials and Methods

### Cell culture

All procedures were approved by the Ethics Committee of the University of Tokyo Hospital (ethics permission #622). Auricular cartilage and costal cartilage were collected from patients with microtia, a congenital deformity where the pinna is underdeveloped, undergoing reconstruction surgery, and knee joint cartilage was collected from patients undergoing artificial joint replacement during surgery in conformity to the Declaration of Helsinki. Chondrocytes were isolated from cartilage and cultivated as reported previously.^12^ Briefly, human chondrocytes were isolated from remnant auricular cartilage or costal cartilage by digestion with 0.15% collagenase (Wako Pure Chemical Industries, Japan). To minimize the risk of contamination with fibroblasts, the perichondrium and cartilage tissue surface of the auricular cartilage were trimmed and discarded, and the remaining auricular cartilage was used for the process. Isolated chondrocytes were seeded on a 100-mm plastic tissue culture dish at a density of 6400 cells/cm^2^ and cultured in DMEM/F-12 (Sigma-Aldrich, St. Louis, MO) containing 5% human serum (Sigma-Aldrich), 100 ng/mL fibroblast growth factor-2 (Kaken Pharmaceutical, Japan), and 5 μg/mL insulin (MP Biomedicals, Irvine, CA) in a 37°C/5% CO_2_ incubator.^13^ Passages were performed by a treatment with trypsin-EDTA solution (Sigma-Aldrich) when cells were approaching 80% confluence. Chondrocytes isolated from auricular cartilage, joint cartilage, and costal cartilage were cultured for eight passages and used to investigate glial fibrillary acidic protein (GFAP) expression. Human dermal fibroblasts, astrocytes, keratinocytes, and airway editorial cells were purchased from Lonza, Inc. (Allendale, NJ). These cells were cultured according to the supplier's protocol.

### Reverse transcriptase–polymerase chain reaction

Total RNA was isolated from cells with Isogen (Nippon Gene, Japan) following the supplier's protocol. Complementary DNA (cDNA) was synthesized from 1 mg of total RNA with the Superscript II reverse transcriptase kit (Invitrogen, CA). Regarding real-time RT-PCR, the ABI Prism Sequence Detection System 7500 was used. Aliquots of first-strand cDNA (1 μg) were amplified with the QuantiTect SYBER Green PCR Kit (Qiagen, The Netherlands) under the following conditions: initial denaturation at 94°C for 10 min, followed by 40 cycles at 94°C for 15 sec, and at 60°C for 1 min. Sequences for the primers are shown in Table 1. Data analyses consisted of fold induction; the expression ratio was calculated from differences in the threshold cycles at which an increase in the reported fluorescence above the baseline signal was initially detected among three samples and was averaged for duplicate experiments.

**Table 1.** List of Primers Used in This Study

Gene	Sense	Antisense
*ACAN*	TCGAGGACAGCGAGGCC	TCGAGGGTGTAGCGTGTAGAGA
*ACTB*	CCAGCACGATGAAGATCAAG	GTGGACAATGAGGCCAGAAT
*COL1A1*	GTGCTAAAGGTGCCAATGGT	CTCCTCGCTTTCCTTCCTCT
*COL2A1*	GAGTCAAGGGTGATCGTGGT	CACCTTGGTCTCCAGAAGGA
*ELN*	CCGCTAAGGCAGCCAAGTATGGA	AGCTCCAACCCCGTAAGTAGGAAT
*GAPDH*	GAAGGTGAAGGTCGGAGTCA	GAAGATGGTGATGGGATTTC
*GFAP*	ACTGTGAGGCAGAAGCTCCA	AGTTCCCGAACCTCCTCCT
*TUBA1A*	ATGGCTTCATTGTCCACCA	GCCCTACAACTCCATCCTGA
*VIM*	GAGAACTTTGCCGTTGAAGC	TCCAGCAGCTTCCTGTAGGT

*ACAN,* aggrecan; *ACTB*, actin beta; *COL1A1*, collagen type I alpha 1 chain; *COL2A1*, collagen type II alpha 1 chain; *ELN*, elastin; *GAPDH*, glyceraldehyde-3-phosphate dehydrogenase; *GFAP*, glial fibrillary acidic protein; *TUBA1A*, tubulin-alpha 1A; *VIM*, vimentin.

### Microarray gene expression profiling

Five micrograms of total RNA from the auricular chondrocyte culture in passage 3 (P3) and P8 was used to direct first-strand cDNA synthesis with the T7-oligo(dT)24 primer and PowerScript reverse transcriptase (Takara Bio USA, Mountain View, CA). After second-strand synthesis and clean-up with the Qiaquick spin column (Qiagen), double-stranded cDNA was used in the MEGA script T7 RNA polymerase *in vitro* transcription reaction (Thermo Fisher Scientific, Waltham, MA), containing the biotin-labeled ribonucleotides, cytidine triphosphate and uridine triphosphate. The resulting labeled cRNAs were hybridized overnight in Affymetrix Gene-Chip Human Genome U133 Plus 2.0 arrays (Affymetrix, Santa Clara, CA), washed, and then scanned according to the manufacturer's instructions using a GeneChip Scanner 3000 and GeneChip Operating Software (GCOS; Affymetrix).

### Differentiation induction of auricular chondrocytes

Auricular chondrocytes cultured at P3 were suspended in 0.8% of atelocollagen solution (Koken, Japan) at a density of 10^7^ cells/mL. In atelocollagen, 20 μL of the cell/material suspension (total 2 × 10^5^ cells) was placed into the bottom of a 15-mL polypropylene conical tube to form a gel in 1-h incubation at 37°C. DMEM/F-12 (Sigma-Aldrich) was used at a volume of 2 mL for each gel and cultured in a 37°C/5% CO_2_ incubator. To induce the differentiation of auricular chondrocytes, 5 μg/mL insulin (MP Biomedicals), 200 ng/mL bone morphogenetic protein-2 (kindly provided by Astellas Pharma, Japan), and 100 nM l-3,3′,5′-triiodothyronine (EMD Bioscience, San Diego, CA) were added to the medium, according to a previous study.^14^ Throughout the experiment, the medium was changed thrice each week.

### Fabrication of tissue-engineered cartilage and transplantation

Poly (l-lactic acid) (PLLA) scaffolds (KRI, Japan), which were produced by the sugar-leaching method,^15^ were used. The molecular weight of scaffolds with an average pore size of 200,000 was 0.3 mm, and average porosity was more than 95%. PLLA scaffolds of 4 × 4 × 3 mm^3^ were sterilized in 70% ethanol before use. A 1% atelocollagen gel, which was diluted from the original 3% atelocollagen gel (Koken) with DMEM/F-12, was then used as a cell suspension buffer to efficiently retain auricular chondrocytes in the scaffolds. To produce tissue-engineered constructs, auricular chondrocytes suspended in the 1% atelocollagen gel (2 × 10^7^ cells/200 μL) were applied to the PLLA scaffolds and incubated at 37°C in 5% CO_2_ for 2 h.

Regarding the transplantation procedure, 8-week-old male mice (C57BL/6; CLEA Japan) were anesthetized by an intraperitoneal injection of sodium pentobarbital (50 mg/kg). A small incision was made on the back in the midline, and constructs were subcutaneously transplanted into each animal. Eight weeks after surgery, the constructs were harvested and cut into equal parts; one piece was frozen in liquid nitrogen and preserved at −80°C for biochemical analyses, while the other piece was fixed in 4% paraformaldehyde for 3 h and used in the histological analysis.

### Quantification of GFAP

Auricular chondrocytes and fibroblasts were cultured with the above methods, and the culture supernatant was harvested 3 and 7 days after passaging. Furthermore, the cell lysate was isolated with M-per (Thermo Fisher Scientific) 7 days after passaging according to the supplier's protocol. The concentration of GFAP was detected using an enzyme-linked immunosorbent assay (ELISA) kit (BioVender, Candler, NC) according to the manufacturer's instructions.

### Histological and immunohistochemical staining

Samples fixed with paraformaldehyde were successively immersed in 10% sucrose phosphate-buffered saline (PBS), 20% sucrose in PBS, and 2:1 mixture of 20% sucrose in PBS and OCT compound (Sakura Finetek, Japan). Samples were rapidly frozen in liquid nitrogen and then cryosectioned at a thickness of 10 μm (CM1850-Kryostat; Leica, Solms, Germany). Sections were stained with toluidine blue-O and observed by optical microscopy (Olympus DP 70, Japan). The sections were also used for immunohistochemical staining for collagen type I (COL1) and collagen type II (COL2).^16^

### Quantification of COL1, COL2, and glycosaminoglycan

Atelocollagen pellet culture and transplanted tissue-engineered cartilage samples were minced using scissors and homogenized twice for 30 sec by a homogenizer (IKA^®^-T10 basic ULTRA-TURRAX^®^). These samples were dissolved in 10 mg/mL pepsin/0.05 M acetic acid at 4°C for 48 h, in 1 mg/mL pancreatic elastase, 0.1 mM Tris, 0.02 M NaCl, and 5 mM CaCl_2_ (pH 7.8–8.0) at 4°C, and were then kept at 4°C overnight. Cell debris and insoluble materials were removed by centrifugation at 6000 *g* for 30 min. Culture supernatant samples were centrifuged at 6000 *g* for 30 min to remove cell debris and insoluble material. We then examined these conditioned samples. The COL1 and COL2 proteins were quantified by ELISA according to the protocol of the human Type I, II Collagen Detection Kit (Chondrex, Redmond, WA). In ELISA measurements, we diluted the supernatant at 1:100 with dilution buffer. In the sample mixture, collagen proteins were captured by polyclonal anti-human COL1 or COL2 antibodies and detected by biotinylated counterparts and streptavidin peroxidase. o-Phenylenediamine dihydrochloride and H_2_O_2_ were added to the mixture, and sulfuric acid was then added after 30 min to stop the reaction. The spectrophotometric absorbance of the mixture was measured at a wavelength of 490 nm by a spectral photometer (ARVO™SX 1420 MULTI LABEL COUNTER; PerkinElmer, Waltham, MA). We evaluated the glycosaminoglycan (GAG) content using the Alcian blue-binding assay (Wieslab AB, Lund, Sweden), according to the supplier's protocol. GAG in the supernatant was precipitated with Alcian blue solution, and sediments after centrifugation at 6000 *g* for 15 min were dissolved again in a 4 M GuHCl-33% propanol solution. The spectrophotometric absorbance of the mixture was measured at a wavelength of 600 nm using a spectral photometer. The collagen or GAG content in the sample was adjusted by the total protein content of the same sample. The total protein content of the sample was measured using the Bio-Rad Dc Protein Assay (Bio-Rad Laboratories, Hercules, CA).

### Immunocytochemistry

Auricular chondrocytes were fixed in 100% ethanol for 15 min. After cells had been washed twice with cold PBS and incubated for 30 min with PBS containing 3% bovine serum albumin (BSA), they were plated on glass slides and incubated at room temperature for 4–6 h with a primary antibody, anti-GFAP (Dako, Glostrup, Denmark), or anti-vimentin (Chemicon, MA), to detect subcellular localization. Secondary antibodies were added to the cells at room temperature for 1 h. Images were acquired using confocal laser scanning microscopy (model TCS-SP5; Leica Microsystems, Heerburgg, Switzerland).

### Flow cytometry

To determine intracellular expression of GFAP, cell suspensions were fixed in 100% ethanol at room temperature for at least 30 min, blocked at room temperature for 1 h in PBS containing 3% BSA, and incubated with the following primary antibody (1:100) at 4°C overnight: anti-GFAP (SC-6170; Santa Cruz Biotechnology, Dallas, TX). After washing with PBS, cells were incubated with the appropriate secondary antibody (1:250) at room temperature for 1 h: mouse anti-rabbit IgG-FITC. Isotype-matched control antibodies (Santa Cruz Biotechnology) were used as negative controls. Fluorescence was measured using a BD LSR-II flow cytometer (BD Biosciences, San Jose, CA) and analyzed with FlowJo Software (BD Biosciences).

### Statistical analysis

All data are presented as mean ± standard deviation. Pearson's correlation was used to assess the relationship among the values of different indexes. A correlation coefficient *r* higher than 0.70 was considered to be a strong correlation; from 0.5 to 0.7 a moderate correlation; and <0.5 a weak correlation.^17^ Statistical significance was evaluated using Bonferroni test for multiple comparisons. A value of *p* < 0.05 was interpreted to denote statistical significance. The statistical program BellCurve for Excel software (Social Survey Research, Inc., Tokyo, Japan) was used for the data analysis.

## Results

### Cultured auricular chondrocytes at early passages strongly express *GFAP* mRNA

**FIG. 1.**
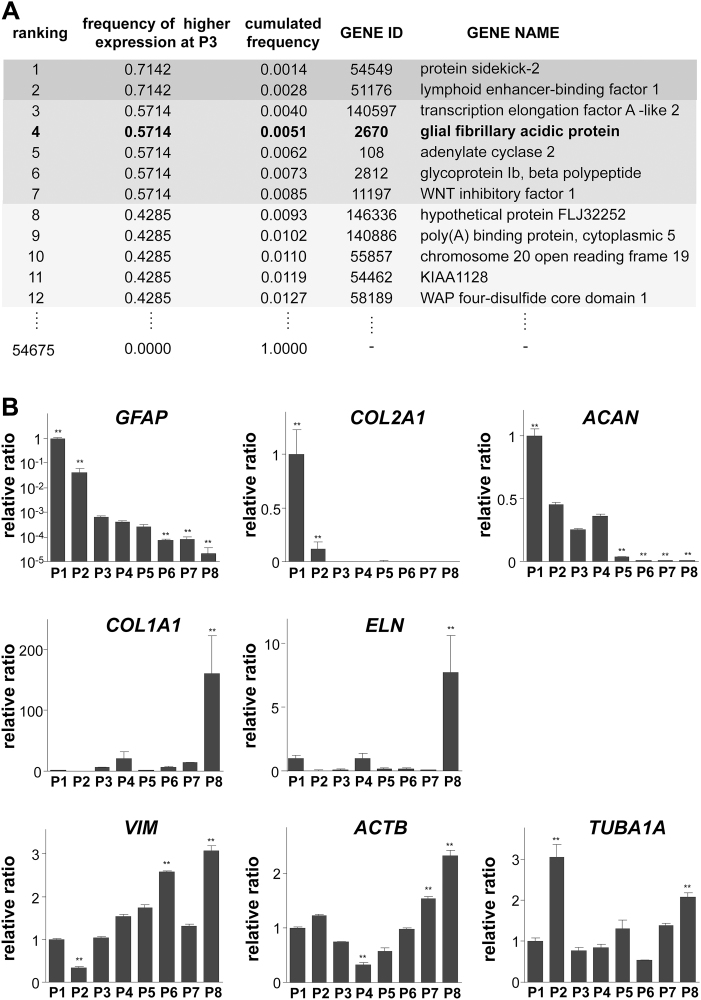
*GFAP* gene expression in auricular chondrocytes decreased with serial passages. **(A)** A microarray analysis of early- and late-passage (passages 3 and 8, respectively) primary human auricular chondrocytes was performed with a Gene_Chip Human Genome U133 Plus 2.0 Array. This analysis was performed on six different donors. The genes that were more strongly expressed at passage 3 were ranked in order of frequency. *GFAP* was ranked fourth out of 54,675 genes. **(B)** Changes in gene expression during serial passages of auricular chondrocytes. Gene expression was evaluated by a real-time reverse transcription–polymerase chain reaction. Each gene expression level was normalized to the level of glyceraldehyde 3-phosphate dehydrogenase, and was shown as a fold difference relative to expression levels at passage 1. Data are shown as mean ± SD for three independent experiments. **p < 0.01 versus P3. ACAN, aggrecan; ACTB, actin beta; COL2A1, collagen type II alpha 1; ELN, elastin; GFAP, glial fibrillary acidic protein; SD, standard deviation; TUBA1A, tubulin-alpha 1A chain; VIM, vimentin.

**FIG. 2.**
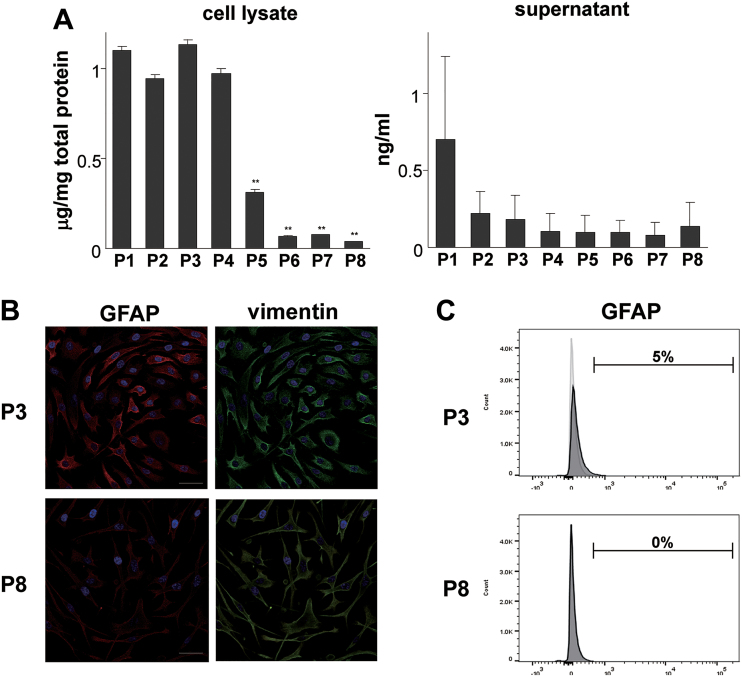
GFAP decreased with serial passages in auricular chondrocyte. **(A)** The amount of GFAP in the culture supernatant and cell lysate of auricular chondrocytes when serially subcultured from passages 1 to 8. Measurements were performed on the seventh day of the culture of each passage. Data are shown as means ± SD for three independent experiments. **(B)** Photomicrographs showing immunofluorescence in passage 3 and 8 auricular chondrocytes stained with vimentin (green) and GFAP (red). Counterstained with 4′,6-diamidino-2-phenylindole (blue). Auricular chondrocytes of passage 8 showed decreased GFAP levels. Scale bars are 10 μm. **(C)** A histogram of flow cytometry showed that GFAP-positive cells decreased in auricular chondrocytes of passage 8. The white histogram represents the negative control staining with fluorescence-conjugated isotype IgG and gray overlay represents antigen at GFAP. **p < 0.01 versus P3.

We examined the expression patterns of more than 47,000 transcripts in the whole genome of human auricular chondrocytes using microarray analyses, and compared them between P3 and P8 of cultured auricular chondrocytes. P3, confirmed to have the characteristics of undifferentiated cells in our previous investigation was selected as undifferentiated auricular chondrocytes, and P8, with the characteristics of dedifferentiation, were selected as early and late passages, respectively.^18^ Expression profiles from the independent samples of six donors were analyzed and had several genes that specifically changed. The expression change in *GFAP* showed a marked decrease at P8 (Fig. 1A). To confirm the decrease in *GFAP* expression at P8 from that at P3, we examined changes in mRNA expression in a long-term culture of human auricular chondrocytes with repeated passages, and the results obtained revealed the marked downregulation of *GFAP* expression (Fig. 1B). The decrease observed in mRNA expression after the long-term culture was also noted in the cartilage-specific matrices, type II collagen (*COL2A1*) and aggrecan (*ACAN*). In contrast, increases were observed in type I collagen (*COL1A1*) and elastin (*ELN*), suggesting the “dedifferentiation” of auricular chondrocytes. Although *GFAP* expression was downregulated with serial passages, the mRNA expression of vimentin (*VIM*), a typical intermediate filament of the mesenchymal cell lineage, actin beta (*ACTB*), a component of the microfilaments and mediator of internal cell motility, and tubulin (*TUBA1A*), the basic structural unit of microtubules, was not downregulated, implying that a decrease in mRNA expression according to the dedifferentiation of auricular chondrocytes was not common in the cytoskeleton, but was specific to *GFAP*. Even at the protein level, *GFAP* was lower at P8 than at P3, which was confirmed by immunostaining. In flow cytometric analyses, GFAP-positive cells were detected in P3, but not detected in P8. These findings strengthened the results of PCR (Fig. 2).

### GFAP distinguishes between auricular chondrocytes and contaminating cells in a primary culture of auricular chondrocytes

**FIG. 3.**
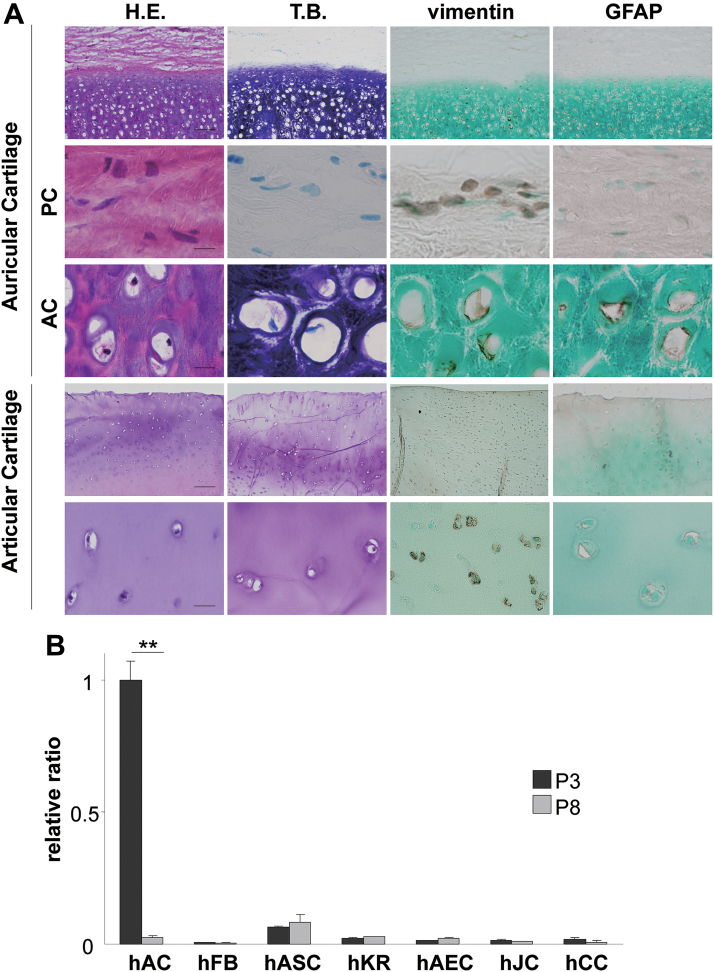
The GFAP expression pattern of auricular chondrocytes is unique. **(A)** Microscopic images of auricular and articular cartilage tissues. GFAP, anti-GFAP immunostain; vimentin, anti-vimentin immunostain. Scale bars were 100 μm (top and fourth stages) and 10 μm (second, third, and bottom stages). **(B)** Gene expression of *GFAP* in various cells cultured to passage 3 (P3) and P8. The expression of *GFAP* was evaluated by a real-time reverse transcription–polymerase chain reaction. Gene expression levels were normalized to glyceraldehyde 3-phosphate dehydrogenase levels and shown as fold differences relative to expression levels at P3 in hAC. Data are shown as mean ± SD for three independent experiments. **p < 0.01 versus P3. AC, auricular cartilage; hAC, human auricular chondrocyte; hAEC, human airway editorial cells; hASC, human astrocytes; hCC, human costal chondrocytes; H.E., hematoxylin and eosin stain; hFB, human fibroblasts; hJC, human articular chondrocytes; hKR, keratinocytes; PC, perichondrium; T.B., toluidine blue stain.

We compared the immunohistochemical findings of auricular and articular cartilage (Fig. 3A). The chondrocytes of auricular cartilage were positive for GFAP and vimentin, while only the latter was localized in the chondrocytes of articular cartilage. Moreover, around auricular cartilage, perichondrial fibroblasts, which are a major cause of cell contamination, were not stained with GFAP. Similar to immunohistochemical findings, *GFAP* expression levels were high in human cultured auricular chondrocytes at P3, while the expression of GFAP was not detected in fibroblasts (Fig. 3B), suggesting that GFAP is a biomarker that distinguishes auricular chondrocytes from perichondrial fibroblasts, one of the most frequent causes deteriorating the purity of auricular chondrocytes during the isolation culture. Using representative GFAP-positive cells, astrocytes, as a positive control, GFAP expression was compared among fibroblasts, keratinocytes, airway editorial cells, auricular chondrocytes, articular chondrocytes, and costal chondrocytes. Surprisingly, the GFAP expression level in P3 astrocytes was markedly lower than that in auricular chondrocytes. GFAP expression was not detected in cells other than astrocytes and auricular chondrocytes (Fig. 3B).

### GFAP is a biomarker that represents the ability of auricular chondrocytes to produce matrix

Since GFAP was specifically expressed in auricular chondrocytes and its expression levels decreased during repeated passages, GFAP may not only be an index that distinguishes auricular chondrocytes from other cells but also a marker that infers matrix production because cartilage matrix production decreases when chondrocytes have excessively proliferated.

Therefore, we investigated whether the measurement time affects the expression level of GFAP.

**FIG. 4.**
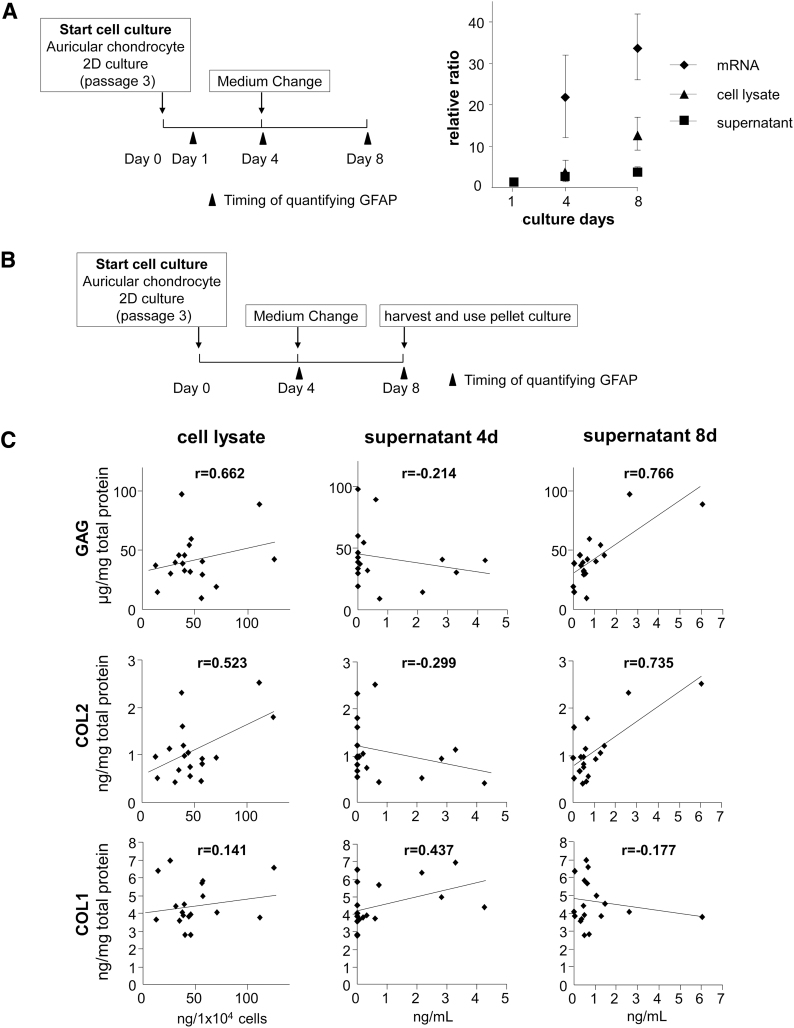
The amount of GFAP in the supernatant of auricular chondrocytes correlates with cartilage matrix productivity. **(A)** Experimental schema for investigating short-term GFAP expression changes in auricular chondrocytes. The amount of GFAP in supernatants and cell lysates and the mRNA expression of P3 auricular chondrocytes were measured on days 1, 4, and 8 after seeding. Each value was expressed as a relative ratio to day 1. Data are shown as mean ± SD for three independent experiments using same lot number of cells. **(B)** Experimental schema to investigate the relationship between the GFAP concentrations of auricular chondrocytes and cartilage matrix productivity. **(C)** Scatter diagram of the GFAP concentrations of auricular chondrocytes and the amount of cartilage matrix accumulated by the induction of differentiation. COL1, type I collagen; COL2, type II collagen; GAG, glycosaminoglycan.

GFAP expression levels were assessed in the same lot of P3 auricular chondrocyte supernatant, cell lysate, and mRNA 1, 4, and 8 days after cell seeding. In all samples, GFAP expression levels increased as the number of culture days became higher. Although gene expression on day 8 was enhanced by more than 30-fold, increases in the protein amount were less in the cell lysate (12-fold) and supernatant (fourfold) (Fig. 4A). These results indicate that if gene expression is used in the quantitative evaluation of GFAP, a few differences in the culture period may result in large variations in the data obtained. If not, the amount of protein may show favorable constancy, particularly in the supernatant. Therefore, we investigated the relationship between the amount of GFAP in the supernatant of auricular chondrocytes and cartilage matrix productivity (Fig. 4B). The amount of GFAP in the supernatant and cell lysate on days 4 and 8 after cell seeding in P3 auricular chondrocytes and the amounts of COL2, COL1, and GAG produced by the induction of auricular chondrocyte differentiation were measured. The amount of GFAP in the cell lysate correlated with the amount of GAG (*r* = 0.662) and that of COL2 (*r* = 0.523). The amount of GFAP in the supernatant on day 4 did not correlate with that of GAG, COL1, or COL2. However, the amount of GFAP in the supernatant on day 8 correlated with the amount of GAG (*r* = 0.766) and COL2 (*r* = 0.735).

### GFAP levels in the supernatant represent the quality of auricular chondrocytes

**FIG. 5.**
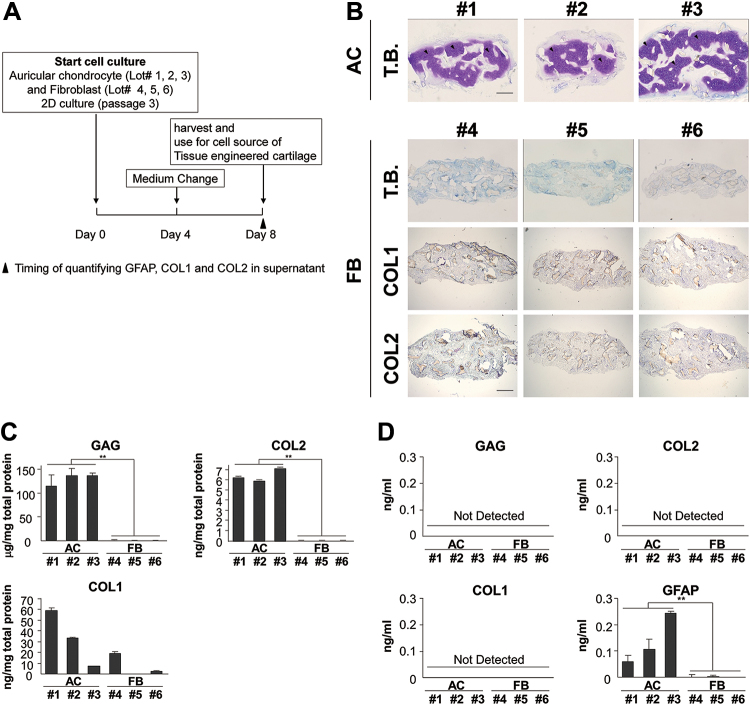
GFAP levels in supernatants may be used to confirm that cultured cells are auricular cartilage cells. **(A)** Time course of this experiment. **(B)** Images of toluidine blue staining and COL1 and COL2 immunostaining of tissue-engineered cartilage prepared with auricular chondrocytes or fibroblasts taken 8 weeks after being transplanted subcutaneously into C57BL6/J mice. Arrow head indicates regenerative cartilage. Area of metachromasia on toluidine blue staining of the auricular chondrocyte construct was shown, while the fibroblast construct did not show any distinctive metachromasia. Immunostaining for COL1 and COL2 revealed the absence of COL1 and COL2 substrates in the fibroblast construct at 8 weeks after transplantation. Scale bars = 1 mm. **(C)** GAG, COL1, and COL2 content of tissue engineered cartilage constructs taken 8 weeks after being transplanted subcutaneously into C57BL6/J mice. **(D)** GAG, COL1, COL2, and GFAP content of the supernatants of auricular chondrocytes and fibroblasts on day 8. **p < 0.01 versus FB group.

In the practical regenerative medicine of cartilage, we need to prevent unsuccessful cases that show the poor regeneration of cartilage. We regarded an unsuccessful case as fibroblasts originating from the perichondrium excessively proliferating to become the main cell population. Therefore, we measured the amount of GFAP in supernatants in which auricular chondrocytes or fibroblasts were seeded at 8 days. Thereafter, these cells were administered into the PLLA scaffold and subcutaneously transplanted into the backs of nude mice, and the contents of type II collagen and GAG were also measured in these tissue-engineered constructs. Auricular chondrocyte-based condition media contained GFAP in all samples (Fig. 5C). The auricular chondrocytes used for GFAP measurements regenerated cartilage after subcutaneous transplantation (Fig. 5A, B). In contrast, GFAP was not detected in the supernatants used for the culture of fibroblasts, which did not result in cartilage regeneration. COL1, COL2, and GAG were not detected in each supernatant. COL1 was slightly positive and COL2 was negative. These findings were not contradictory to the results of protein quantitation shown in Figure 5B and C.

## Discussion

This study confirmed the following findings on GFAP in auricular chondrocytes. Auricular chondrocytes express GFAP; however, its expression level decreases when they are dedifferentiated. These changes in GFAP levels may be detected in mRNA, cell lysates, and culture supernatants. However, GFAP levels in the mRNA and cell lysates of auricular chondrocytes markedly varied with the culture duration for a specific passage, whereas those in culture supernatants were affected less. GFAP levels in the culture supernatant of auricular chondrocytes correlated with the cartilage matrix productivity of differentiation-induced cells. Accordingly, the cartilage matrix productivity of cultured auricular chondrocytes may be predicted by measuring GFAP levels. GFAP levels in the culture supernatant of auricular chondrocytes may be applied to the monitoring of quality auricular chondrocytes as a cell source of regenerative cartilage.

Since GFAP is specifically expressed in the cells of astrocyte linage, it is widely used as an astrocyte marker in the brain. The serum GFAP concentration is used as a biomarker to diagnose intracranial and spinal tumors caused by astrocytes.^19,20^ The diagnostic method for traumatic brain injury using GFAP in the serum as an index shows high detection sensitivity and is approved by the FDA.^21^ We considered that GFAP, which has established clinical-level testing methods, could be applied as a useful marker in auricular chondrocytes.

In the long term, GFAP mRNA expression in auricular chondrocytes decreased in passage culture. This phenomenon was also confirmed in the GFAP protein level in cell lysate and culture supernatant. In addition, similar results were acquired by immunostaining and flow cytometry analysis. However, protein detection showed a mild decrease compared with the drastic decrease of mRNA. This finding was consistent with a reported finding that there was only a weak correlation between the mRNA and protein levels.^22^ In the short term, GFAP increased within a several-day period. This change was marked in mRNA and cell lysate. It is known that GFAP expression makes a large variation between before and after mitosis, suggesting that the cell growth rate influences GFAP expression.^23^ Since the cell growth rate markedly changes within a short term after initiation of culture, it may be necessary to take this influence into consideration when GFAP expression is compared. The optimum timing of GFAP measurement may be the time point when cultured cells reach 80% confluence because the cell growth rate reaches a peak at this time point so that the conditions can be easily uniformed among experiments. Cell adhesion is unstable for 1–2 days after seeding and many cells are in the resting phase of the cell division cycle, which may be inappropriate for GFAP measurement. Moreover, the cell growth rate sharply increases in the period from day 2 to reaching 80% confluence, so that it is difficult to uniform the conditions among experiments. In contrast, the short-term changes in the GFAP level in the culture supernatant were small. Since the culture supernatant is a sample of cumulative GFAP expression of cells cultured for several days, it is unlikely to be influenced by short-term change in the expression level. However, the GFAP level in the culture supernatant showed a large variation in the supernatant of passage 1. Since passage 1 was immediately after isolation from the body, cells were not acclimated to the *in vitro* culture conditions and this may have influenced the GFAP level.

The presence of large variations in the cartilage matrix productivity of cultured auricular chondrocytes is a major issue in the manufacture of regenerated cartilage because it may contribute to the contamination of auricular chondrocytes with low cartilage productivity.^24,25^ Therefore, we need to develop a simple method to evaluate the cartilage matrix productivity of auricular chondrocytes to facilitate the realization of treatment with regenerated cartilage. One candidate method to evaluate the characteristics of cultured cells is real-time PCR or ELISA with cell lysate samples.^26,27^ However, the findings of real-time PCR are presented as relative values and their standardization is difficult.^28,29^ In contrast, ELISA is more appropriate for standardization because measurement results are presented as absolute values.^30,31^ ELISA is clinically employed in biochemistry tests and applied to the diagnosis and judgment of treatment.^32,33^ Thus, we considered a search for a protein index of auricular chondrocytes to be reasonable.

ELISA is widely used to measure the concentrations of various proteins contained in cells.^4,34^ However, cell sampling is necessary for measuring protein levels in cells, implying that some cells will be lost to the measurement. On the other hand, the culture supernatant is normally discarded when culture medium is exchanged. Thus, if it is possible to evaluate and detect a specific protein in the culture supernatant, it will be superior to tests using cell samples because cells will not be lost. This will be very useful in the field of regenerative medicine in which valuable donor-derived cells are the material.

We analyzed the correlation between the measured GFAP levels in the culture supernatants and cell lysates on days 4 and 8 corresponding to 30% and 80% confluence, respectively, and matrix productivity of auricular chondrocytes. A correlation was noted in the culture supernatant of day 8. This finding strengthened the hypothesis concerning appropriate timing and measurement described above. In the *in vivo* test, we could confirm that cultured cells were auricular chondrocytes with the characteristics of undifferentiated cells by detecting GFAP in the culture supernatant on day 8 in the 2D passage culture process before transplantation. This result may be beneficial because the quality of regenerated cartilage can be judged before transplantation.

GAG, COL1, and COL2 were not detected in the culture supernatant and this may have been due to a level in the culture supernatant lower than the detection sensitivity and it may be improved by developing a measurement method with high sensitivity. In the transplanted fragment using fibroblasts as a cell source, accumulation of the major component of fibroblasts, GAG, could not be detected. One reason for this may have been early dying out of fibroblasts because fibroblasts were transplanted under the administration conditions (number of cells, type and concentration of atelocollagen, and pore size of PLLA) optimized for auricular chondrocytes. Other may be because matrix production of fibroblasts in fibrous tissue is principally much less compared with chondrocytes in cartilaginous tissue. Although we had reported in another article,^35^ we performed GFAP over expression or downregulation of auricular chondrocytes and confirmed whether the cartilage matrix productivity changed. In addition, we performed an experiment comparing the cartilage matrix productivity of auricular chondrocytes in GFAP knockout mice with that in wild-type mice, but GFAP expression did not influence the deposition of the cartilage matrix in either experiment. GFAP serves as an indirect index of undifferentiated auricular chondrocytes, but it has no direct action on the process of deposition of the cartilage matrix.

We set a 0.05 ng/mL or higher GFAP level in the culture supernatant of auricular chondrocytes on day 8 as a quality monitoring criterion of auricular chondrocytes to be used as a cell source of implant-type tissue-engineered cartilage. Then, we produced implant-type tissue-engineered cartilage and performed clinical trials in three patients.^36^ The treatment showed efficacy at 1 year after transplantation in all patients, suggesting that GFAP in the auricular chondrocyte culture supernatant is useful as a quality index. This criterion is adopted for quality control in an ongoing clinical study aiming at industrialization.

## Abbreviations Used

AC: auricular chondrocytes
ACAN: aggrecan
ACTB: actin beta
AEC: airway editorial cells
ASC: astrocytes
BSA: bovine serum albumin
CC: costal chondrocytes
cDNA: complementary DNA
COL: collagen
COL1A1: collagen type I alpha 1 chain
COL2A1: collagen type II alpha 1 chain
DMEM/F-12: Dulbecco's modified Eagle's medium: nutrient mixture F-12
EDTA: ethylenediaminetetraacetic acid
ELISA: enzyme-linked immunosorbent assay
ELN: elastin
FB: fibroblast
FDA: Food and Drug Administration
GAG: glycosaminoglycan
GAPDH: glyceraldehyde-3-phosphate dehydrogenase
GFAP: glial fibrillary acidic protein
H.E.: hematoxylin and eosin
JC: articular chondrocytes
KR: keratinocytes
PBS: phosphate-buffered saline
PC: perichondrium
PLLA: poly (l-lactic acid)
RT-PCR: reverse transcriptase–polymerase chain reaction
SD: standard deviation
T.B.: toluidine blue
TUBA1A: tubulin-alpha 1A chain
VIM: vimentin
